# Impact of social isolation during COVID-19 on anthropometric data, quality of life, baseline physical activity and aortic pulse wave parameters in children and adolescents in two independent samples

**DOI:** 10.1186/s13052-023-01558-w

**Published:** 2023-11-19

**Authors:** Mariana Godoy-Leite, Fernanda Gabriela Colombo Drumond Santos, Eduardo Augusto Resende Penido, Kennad Alves Ribeiro, Luzia Maria dos Santos, Maria da Gloria Rodrigues-Machado, Bruno Almeida Rezende

**Affiliations:** School of Medical Sciences of Minas Gerais, 275, Alameda Ezequiel Dias, Belo Horizonte, 30130-110 Brazil

**Keywords:** Arterial stiffness, Pulse wave velocity, COVID-19, Quality of life

## Abstract

**Background:**

The social restrictions resulting from the COVID-19 pandemic had a great impact on the routine of children and adolescents, with important consequences such as sleep, eating, and psychological/psychiatric disorders. Even though there are no studies on the subject, it is possible that these changes in habit and routine have also affected arterial stiffness (AS) in this population, which is an important predictor of cardiovascular risk. This study aimed to assess possible changes in AS, anthropometry, and quality of life (QoL) resulting from the COVID-19 pandemic in children and adolescents.

**Methods:**

A controlled observational cross-sectional study was performed with 193 children and adolescents aged 9 to 19 years, allocated into two groups: before the pandemic (BPG) and one year after the pandemic (APG), matched by age and sex. Cardiovascular parameters were measured non-invasively by brachial artery oscillometry with a portable device. The main AS indices evaluated were the augmentation index (AIx) and pulse wave velocity (PWV) derived from the aortic pulse wave. QoL was assessed using the Paediatric Quality of Life Inventory version 4.0 (PedsQL 4.0).

**Results:**

Regarding QoL, the APG showed a worsening in emotional (p = 0.002) and school-related (p = 0.010) aspects. There was no statistically significant difference for most anthropometric parameters, except for the hip circumference, which was higher in the APG group (p < 0.001). The main predictor of AS in the paediatric population, AIx@75, was shown to be increased in the APG group (p < 0.001). Other cardiovascular parameters were also different, such as peripheral (p = 0.002) and central (p = 0.003) diastolic blood pressure, stroke volume (p = 0.010), and total vascular resistance (p = 0.002), which were shown to be decreased in the APG group, while the heart rate was increased (p < 0.001).

**Conclusions:**

Our results show that routine changes resulting from the period of social isolation increased cardiovascular risk in children and adolescents, evident by the increase in AIx@75, which is considered to be an important marker of cardiovascular risk in the paediatric population.

## Introduction

The COVID-19 pandemic, which began in March 2020, is considered a global health crisis that, in addition to having resulted in millions of deaths, has had social and financial consequences for people around the world [1]. With the uncontrollable advance of the disease, several countries have adopted strategies to contain its transmission due to the impossibility of adopting accessible and effective treatments [2].

According to data from the United Nations International Children’s Emergency Fund (UNICEF), approximately 2.34 billion children have been affected by restrictions in 186 countries [1]. Thus, the norms of social isolation exposed children and adolescents to biopsychosocial stressors that became an adverse experience of childhood and adolescence, bringing potential damage to individual and collective health, with probable short- and long-term repercussions [3]. The impossibility of face-to-face classes, reduction in physical and leisure activities, children’s sedentary lifestyle, and the time of exposure to screens, compromised the mental health of children and adolescents [4]. Some studies already point out important consequences of this change in routine, such as sleep, eating, and psychological/psychiatric disorders [5].

Evidence shows that cardiovascular diseases, such as hypertension, are increasing among young people. This likely relates to obesity and physical inactivity, conditions that have intensified during the COVID-19 pandemic [6]. In a systematic review, the authors provide information and raise awareness about the impact of the COVID-19 pandemic on the prevalence of risk factors for acquired atherosclerotic cardiovascular disease (ASCVD) in adolescents due to changes in usual lifestyle that culminated in physical inactivity, excessive weight gain, insulin resistance/diabetes and dyslipidemia, commonly associated with ASCVD. Early identification and optimal management of CVD risk factors play an important role in preventing future cardiovascular disease [7], as well as, to contribute to the development of strategies to help families deal with the adversity caused by the epidemic/pandemic and ensure the healthy development of their children.

In addition, all the stress generated due to the uncertainties related to the pandemic can also have diverse repercussions on the homeostasis of various systems, including the cardiovascular system. Stress is usually approached from a medical or biological point of view, and it is well documented in the scientific literature that childhood stressors are related to increased cardiovascular morbidity in adulthood [8] and centralisation of body fat [9]. Similar to adults, traditional risk factors including perinatal history, a family history of hypertension, obesity, minority race/ethnicity, physical inactivity, and a high dietary intake of sodium are associated with an increased risk of elevated BP and hypertension [10]. Epidemiological studies show an increased incidence of arterial hypertension in childhood, probably due to the association of overweight, obesity, and physical inactivity, which were even more frequent and evident in the period of COVID-19 [6]. Song et al., in a sensitive analysis showed that the pooled prevalence of hypertension among children varied from 3.85% [11].

In addition to the impact of restrictive measures, we also have the possibility of direct changes promoted by SARSCoV-2 on cardiovascular health. Although we don’t have direct studies on the direct impact of SARS-CoV-2 infection on arterial stiffness in children and adults, the CARTESIAN study proposes to investigate the impact of COVID-19 infection in arterial stiffening [12]. What is known so far is that SARS-CoV-2 can directly infect endothelial cells, inducing marked endothelial damage and inflammation [13]. Furthermore, Kawasaki-like inflammatory syndrome, children’s most common primary vasculitis, its known to be associated with aortic stiffness and has been described after COVID-19 infection [14]. In addition, Szekely et al. have reported diastolic dysfunction in 16% of patients after COVID-19. This could be related to alterations in small vessels and capillaries since there is a direct relation between the micro-circulation and cardiac diastolic dysfunction [15].

The increase in blood arterial pressure in young patients is related to signs of premature vascular ageing, which is explained by the appearance of structural alterations in the arterial vessels, such as arterial stiffness [9]. This term characterises dysfunctions in the physical properties of arterial walls, such as distensibility and compliance, and is related to the process of premature atherosclerosis [16]. It is clear, therefore, that vascular disease can start early in childhood and remain asymptomatic, in a subclinical way, until it manifests itself in adulthood [16]. Coronary artery disease, for example, begins very early in life, albeit subclinically, and precedes the findings of atherosclerotic lesions since the compliance of the large arteries, which are rich in elastic fibres, is intact in young populations with normal blood arterial pressure [16]. Thus, the stiffness of the great arterial vessels has been considered as an independent non-invasive marker of cardiovascular risk [16]. Currently, efforts have been made to find early markers of cardiovascular disease so that timeous intervention can be carried out to reduce the development and progression of cardiovascular diseases, thus highlighting the importance of investigating arterial stiffness [17]. Possible early cardiovascular risk markers include pulse wave velocity (PWV), considered a great indicator of arterial stiffness [18], and the augmentation index normalized to a heart rate of 75 beats per minute (AIx@75), an independent predictor of future cardiovascular events and mortality [19, 20].

The purpose of this study was to evaluate cardiovascular, anthropometric, and quality of life (QoL) parameters in children and adolescents, one year after the implementation of restrictive measures against the COVID-19 pandemic, and to compare them to a database obtained in the period before the pandemic. The opportunity to study a population that spent more than one year in unusual conditions may help to understand the cardiovascular and metabolic impact of routine change in children through these variables.

## Materials and methods

### Study design

This is a cross-sectional, observational study that compared cardiovascular and anthropometric parameters and the impact on the QoL of healthy children and adolescents, after more than one year of the pandemic, with a control group matched by age, sex, and socioeconomic class, evaluated in a pre-pandemic period.

### Participants

A total of 193 children and adolescents participated in this study, allocated into two groups: Pre-Pandemic (Before the Pandemic Group - BPG) and Post-Pandemic (After the Pandemic Group - APG). BPG consisted of 89 participants from a database consisting of children and adolescents of both sexes aged 9 to 19 years, who were students from public schools in the city of Belo Horizonte, Minas Gerais, Brazil and who participated in a previous study by Santos et al. [19]. Cardiovascular, anthropometric, and QoL parameters were evaluated between June 2016 and March 2017. APG consisted of 104 children and adolescents, matched by age, sex, and socioeconomic class, from public schools in the same location. Data were collected after the period of more than one year after the beginning of the COVID-19 pandemic (August to October 2021).

The socioeconomic class was determined as recommended by ABEP (Brazilian Association of Research Companies) [21]. This classification is based in a questionnaire to evaluate the ownership of assets. For each asset owned there is a score, and each socioeconomic category is defined by the sum of this score. After defining the classes, ABEP estimates an average monthly income per family as shown below: A:45–100 pts/R$ 20,888.00; B1: 38–44 pts/R$ 9254.00; B2: 29–37 pts/R$ 4852.00; C1: 23–28 pts/R$ 2409.01; C2: 17–22 pts/R$ 1625.00; D–E: 0–16 pts/R$ 768.00. Subsequently, to obtain greater statistical power, we combined classes for a final total of five classes: A, B (B1 + B2), C (C1 + C2), D and E.

In Belo Horizonte, schools remained closed throughout March 2020 to July 2022. In addition, parks, shopping malls and churches were also closed during this period, with drastic restrictions on socializing the population. In August 2021, public schools in Belo Horizonte created a protocol for the partial resumption of classes for high school and elementary school students. This protocol recommended that each child could attend one week per month of face-to-face classes in schools. Thus, it was possible to collect data for the APG group during this period. Although the schools involved in the study were the same, no participant from the BPG group was included in the APG group.

Volunteers who reported acute or chronic, respiratory, cardiovascular and/or renal diseases, diabetes, smoking history, and systemic arterial hypertension were excluded. To investigate possible respiratory diseases, the International Study of Asthma and Allergies in Childhood (ISAAC) questionnaire was used, excluding volunteers with a score greater than or equal to 5 points [22]. These conditions require exclusion from the study as they can directly interfere with the results of the cardiovascular assessment [23–27]. Volunteers who reported having been previously diagnosed with COVID-19 infection were also excluded.

### Anthropometric assessment

Weight, height, and waist and hip circumferences (WC and HC) were evaluated. Waist-to-hip (WC/HC) and waist-to-height ratios (WHtR) were also calculated. The World Health Organization (WHO) considers the WC/HC as a criterion to characterise metabolic syndrome and cardiovascular risk, with cut-off values of 0.90 for men and 0.85 for women [28]. Also, for Brazilian population, the BMI and WC are considered the main predictors of metabolic syndrome in children (cut-off values of 78 cm for WC, and 24.5 kg/m^2^ for BMI) [29].

### Assessment of baseline physical activity level

The short version of the Physical Activity Questionnaire-Child was used to assess habitual physical activity level, which investigates the practice of physical activity in children and adolescents in the seven days prior to filling it out. The IPAQ-A and IPAC-C were used for children and adolescents, respectively. It consists of nine questions related to physical activity performed at school and during leisure time during the week and on weekends. Each question has scores from 1 to 5. Those who spent 150 min performing moderate physical activities during the week were considered active [30].

### QoL assessment

Participants’ QoL was assessed using the Paediatric Quality of Life Inventory version 4.0 (Peds-QL 4.0). This questionnaire consisting of 23 questions that assess the perception of children and adolescents and is divided into two domains: physical (8 items), emotional—cognitive and intellectual (5 items), social (5 items), and school (5 items). Items were rated on a scale of points from zero to 100; where zero = 100 points; one = 75; two = 50; three = 25; four = 0. Then, the average of these items was measured. The higher the score, the better the perception of quality of life. [31]. This questionnaire was answered directly by the research participants and, when necessary, assistance was provided by the researchers involved in the study.

### Assessment of cardiovascular parameters

Arterial stiffness was estimated by measuring PWV and AIx@75, using the Mobil-O-Graph® instrument (IEM Germany-The Pulse Wave Analysis Monitor, version 4.8, Vienna, Austria), according to Santos et al. [19].This equipment uses an oscillometric method of assessing brachial blood pressure that yields a non-invasive estimation of central blood pressure. The shape of the aortic pressure wave was obtained by summing the incident pressure waves generated by ventricular contraction and the reflected pressure wave coming from the periphery. The augmentation pressure (AP), corresponding to the increase in SBPc due to the reflection wave, expressed as a percentage of central pulse pressure (PPc), corresponds to AIx@75 = AP/PPc × 100. The ARCSolver algorithm allows the PWV to be calculated using a mathematical model, considering several parameters in the pulse wave and the wave separation analysis [32].

The device also allowed the evaluation of a series of other parameters such as peripheral systolic (pSBP) and diastolic (DBPp) blood pressures, peripheral mean arterial pressure (MAPp), and peripheral pulse pressure (PPp), as well as central diastolic (DBPc) and systolic (SBPc) blood pressures, and central pulse pressure (PPc). In addition, stroke volume (SV), cardiac output (CO), cardiac index, total vascular resistance (TVR), and heart rate (HR) were also obtained.

Similar to the cardiac index, which allows comparison between different individuals, the relationship between SV and body surface area expressed in square metres was used.

The classification of SBPp and SBPp were performed according to the recommended percentile of each age group. A blood pressure less than the 90th percentile is considered normal; between the 90th and 95th percentile designates prehypertension. For adolescents’ population, blood pressure equal to or exceeding 120/80 mmHg denotes prehypertension, even if this figure is less than the 90th percentile. This classification is in accordance to the recommendations of the National Institutes of Health National Heart and the VI Brazilian Guidelines for Hypertension [33, 34]. Therefore, a new categorization of normal and elevated blood pressure was defined by age, height, and sex according to the percentile.

Cardiovascular parameters were performed according to previous studies by our group [19, 20, 24, 35]. The measurements were performed with the participant sitting, in a calm and quiet place, after a minimum rest of 5 min. The cuffs were selected according to the circumference of the participant’s left arm, where the measurements were taken. The room temperature was maintained between 21 degrees according to the manufacturer’s recommendation. The measurements were performed in triplicate and the average of three acceptable was considered for the final analysis of all parameters evaluated. The Mobil-O-Graph can also be implemented as an ambulatory PWV measuring tool for cardiovascular risk stratification in children and adolescents since its has been validated for paediatric population [36, 37].

### Sample size

The sample size was calculated to test the difference in the means of AIx@75 of children and adolescents in the pre- and post-pandemic period [38]. To obtain a significance level of 5% and minimum power of 80%, and to test a minimum difference of 4.1 in AIx@75 in relation to the mean of AIx@75 obtained in a previous study [16], at least 60 individuals were needed in each group.

The formula used for the sample calculation was:

$$n=2 {\left(\sigma \frac{{z}_{1-\alpha }+{z}_{1- \beta }}{{\mu }_{a}-{\mu }_{b}}\right)}^{2} \sigma =\text{11,5} {\mu }_{a}=\text{79,6} {\mu }_{b}=\text{85,6}$$

### Statistical analysis

Qualitative variables were presented as frequencies and quantitative variables as mean ± standard deviation (median). Quantitative variables were submitted to the Shapiro-Wilk normality test. The association between qualitative variables was assessed using the Chi-Square test. For the comparison of quantitative variables between two groups, the Student’s t-test was used for independent samples, as well as the Wilcoxon Mann-Whitney test. The analyses were developed in the R program version 4.0.5 and p < 0.05 was considered significant.

## Results

**Fig. 1:**
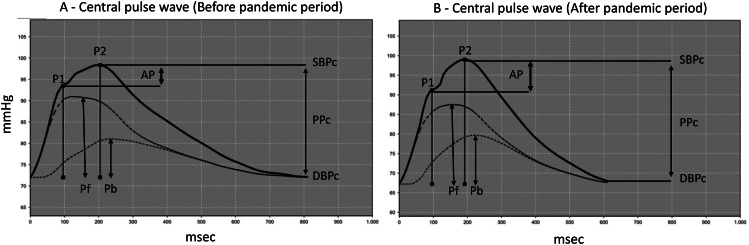
Pulse waves from the aortic arteries (APW). **A**- APW before pandemic period. **B**-APW after pandemic period. P1 = First systolic peak; P2 = Second systolic peak; Pf = Forward or ejection wave; Pb = Backward or reflection wave; SBPc and DBPc = central systolic and diastolic blood pressures; PPc = central pulse pressure. AIx@75=(P2-P1)/cPP*100 corrected for a heart rate of 75 bpm. AIx@75 = 19% and AIx@75 = 37% in A and B, respectively

**Fig. 2:**
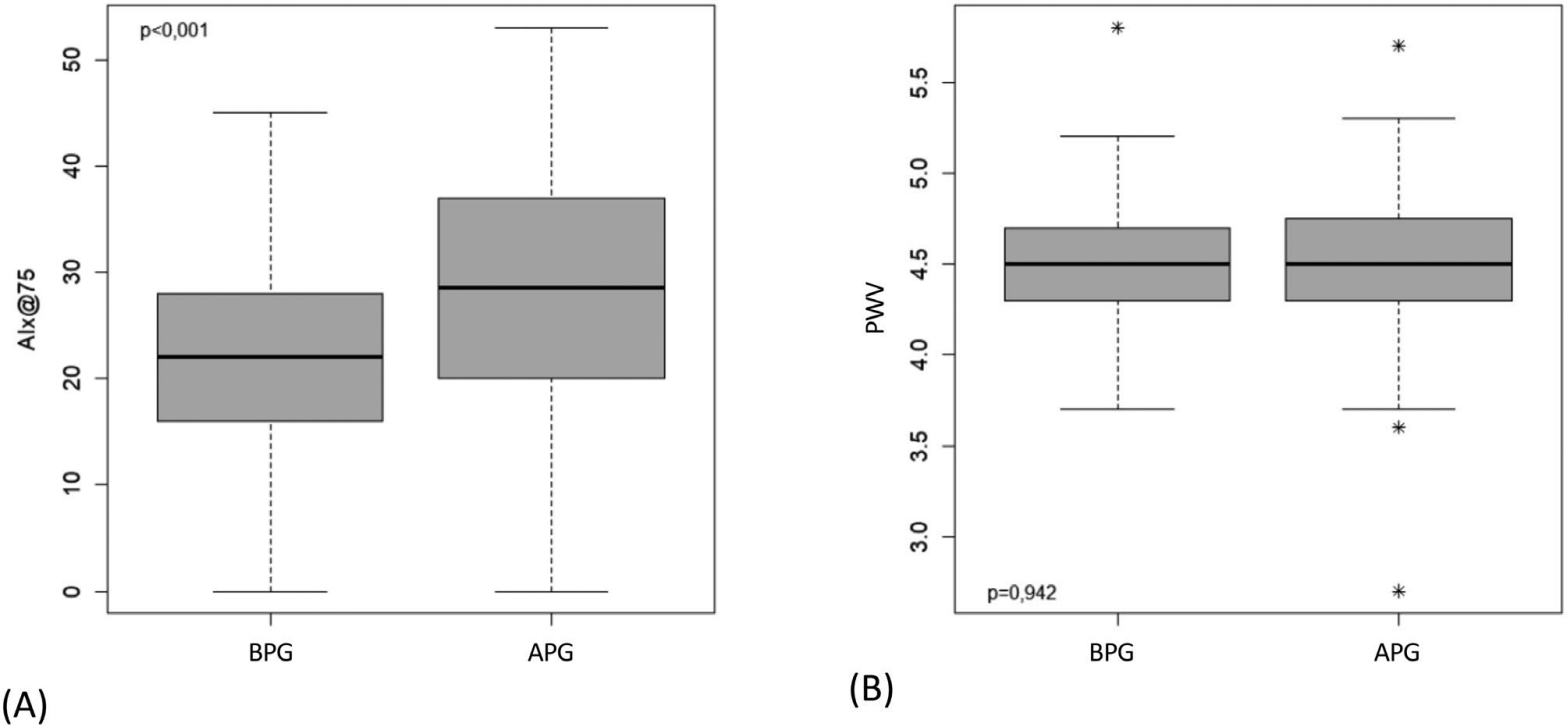
Box plot representing the evaluation of arterial stiffness parameters in the groups before (BPG) and after (APG) the pandemic: (**A**) augmentation index corrected for heart rate of 75 bpm (AIx@75%); (**B**) pulse wave velocity (PWV m/s). The p-values refer to the Wilcoxon Mann-Whitney test

The sample consisted of 193 children and adolescents, aged between 9 and 19 years (mean age 12.1 ± 2.8 years), 51.8% of which were female. In the BPG, 89 cases (46.1%) were evaluated, and in the APG, 104 cases (53.9%). The BMI was 19.4 ± 3.6 kg/m^2^, and the mean value of WC was 71.6 ± 11.8 cm. Regarding the profile of habitual physical activity, 57.51% were sedentary. More than half, 67.4%, were from classes C, D, or E. As for quality of life, the physical and social aspects presented means over 80 (84.3 ± 13.6 and 86.1 ± 15.7, respectively), while the emotional and school aspects presented means greater than 70 (72.8 ± 20.1 and 77.8 ± 17.1, respectively). The individuals evaluated in the APG had higher mean values of HC (p < 0.001), and lower values of QoL in the emotional (p = 0.002) and school (p = 0.010) aspects (Table 1). The children and adolescents evaluated in the APG had higher values of HR (p < 0.001), PPp (p = 0.006), and AIx@75 (p < 0.001), and lower values of DBPp (p = 0.002), DBPc (p = 0.003), SV (p = 0.010), and TVR (p = 0.002) (Table 2). Figure 1 shows the central pulse wave evaluated in participants of the BPG and APG and Fig. 2 presents the boxplot of the main arterial stiffness markers in both groups.

**Table 1:** Sociodemographic characterisation, physical activity profile, and quality of life of the individuals in the sample, according to the evaluation period (pre- and post-pandemic)

Parameters	BPG (n = 89)	APG (n = 104)	P-value
Gender			0.182^Q^
Female	41 (46,1%)	59 (56,7%)	
Male	48 (53,9%)	45 (43,3%)	
Age (years)	12,3 ± 3,0 (12,0)	12.2 ± 2.6 (11,0)	0.684^ W^
Weight (kg)	46.1 ± 11,2 (45,8)	48.3 ± 16.0 (46,2)	0.757^ W^
Height (cm)	154.7 ± 13,3 (156,0)	154.4 ± 13.7 (153,0)	0.640^ W^
BMI (kg/m^2^)	19.0 ± 2,4 (18,8)	19.8 ± 4.4 (19,0)	0.514^ W^
WC (cm)	72.6 ± 11,5 (73,0)	70.7 ± 12.0 (69,0)	0.072^ W^
HC (cm)	77.0 ± 13,0 (74,5)	84.3 ± 12.3 (83,3)	**< 0.001** ^** W**^
WC/HC	0.9 ± 0,2 (0,8)	0.9 ± 0.1 (0,8)	0.468^ W^
WHtR	0.5 ± 0,1 (0,4)	0.5 ± 0.1 (0,4)	0.781^ W^
IPAQ-C			0.097^Q^
Activities	44 (49,4%)	38 (36.5%)	
Sedentary	45 (50,6%)	66 (63.5%)	
Quality of Life			
Physical	85.2 ± 11,7 (84,4)	83.6 ± 15,0 (87.5)	0.795^ W^
Emotional	77.4 ± 18,6 (80,0)	68.9 ± 20,6 (70.0)	**0.002** ^** W**^
Social	88.2 ± 12,4 (90,0)	84.3 ± 18,0 (90.0)	0.413^ W^
School	81.5 ± 14,9 (85,0)	74.5 ± 18,2 (75.0)	**0.010** ^** W**^
Socioeconomic categories according to ABEP			1.000^Q^
A or B	29 (32.6%)	34 (32.7%)	
C, D, or E	60 (67.4%)	70 (67.3%)	

Data were expressed as mean ± standard error of the mean (median). Post-pandemic group (APG), pre-pandemic group (BPG), waist circumference (WC), hip (HC), waist-to-height ratio (WHtR), body mass index (BMI), childhood physical activity questionnaire (IPAQ-C). ABEP, Associação Brasileira de Empresas de Pesquisa. Q: Chi-Square test, W: Wilcoxon Mann-Whitney test

**Table 2:** Measurements of peripheral and central blood pressure, hemodynamic parameters, and arterial stiffness of the individuals in the sample, according to the evaluation period (pre- and post-pandemic)

Variables	BPG (n = 89)	APG (n = 104)	P-value
Peripheral blood pressure			
SBP (mmHg)	111.7 ± 10.3 (112.0)	111.6 ± 11.5 (110.0)	0.918^ W^
DBP (mmHg)	69.6 ± 7.3 (70.0)	65.7 ± 9.4 (66.0)	**0.002** ^**T**^
MBP (mmHg)	88.7 ± 7.5 (87.0)	86.7 ± 8.7 (87.0)	0.088^ W^
PP (mmHg)	41.7 ± 9.4 (41.0)	45.9 ± 11.4 (44.0)	**0.006** ^** W**^
Central blood pressure			
SBP (mmHg)	99.6 ± 8.5 (99.0)	98.2 ± 10.4 (97.0)	0.324^ W^
DBP (mmHg)	71.3 ± 7.3 (71.0)	67.7 ± 9.4 (68.0)	**0.003** ^**T**^
PP (mmHg)	28.4 ± 6.3 (28.0)	30.5 ± 8.4 (29.0)	0.110^ W^
Haemodynamics			
SV (ml)	57.2 ± 13.4 (55.4)	52.3 ± 12.9 (50.1)	**0.010** ^** W**^
CO (l/min)	4.4 ± 0.6 (4.3)	4.5 ± 0.7 (4.5)	0.086^T^
TVR (s*mmHg/ml)	1.24 ± 0.14 (1.23)	1.17 ± 0.16 (1.17)	**0.002** ^**T**^
CI (l/min*l/m^2^)	3.2 ± 0.7 (3.1)	3.3 ± 0.6 (3.1)	0.847^ W^
HR (bpm)	78.1 ± 12.7 (78.0)	87.2 ± 14.0 (87.0)	**< 0.001** ^** W**^
Arterial Stiffness AP (mmHg) PPA RC (%)	6.7 ± 4.0 (1.2) 1.5 ± 0.2 (1.4) 58.0 ± 10.4 (25.0)	6.1 ± 2.7 (1.2) 1.5 ± 0.2 (1.5) 57.03 ± 10.1(22.0)	0.259 ^W^ 0.680^ W^ 0.505 ^W^
AIx@75 (%)	22.2 ± 9.4 (22.0)	27.6 ± 11.4 (28.5)	**< 0.001** ^** W**^
PWV (m/s)	4.5 ± 0.3 (4.5)	4.5 ± 0.4 (4.5)	0.942^ W^

Data were expressed as mean ± standard error of the mean (median). Post-pandemic group (APG), pre-pandemic group (BPG), systolic (SBP), diastolic (DBP) blood pressures, mean arterial pressure (MAP), and pulse pressure (PP), pulse pressure amplification (PPA), augmentation pressure (AP), stroke volume (SV), cardiac output (CO), cardiac index (CI), total vascular resistance (TVR), heart rate (HR), reflection coefficient (RC), pulse wave velocity (PWV), augmentation index normalized by 75 bpm (AIx@75).

W: Wilcoxon Mann-Whitney test, T:0 Student’s t-test for independent samples

## Discussion

This study evaluated whether changes in the routine of children and adolescents during the period of social isolation resulting from the COVID-19 pandemic had any impact on anthropometric data, quality of life, and possible subclinical changes suggestive of arterial stiffness in this population. To the best of our knowledge, this is the first controlled study where cardiovascular variables and QoL were compared in similar populations in the pre- and post-pandemic periods. This study demonstrated that the main subclinical marker of arterial stiffness in the paediatric population, represented by AIx@75, which is a highly predictive variable of cardiovascular events [26], was increased in children and adolescents after one year of social isolation.

The period of social isolation resulting from the COVID-19 pandemic can be considered a toxic childhood environment due to the various negative changes that occurred during the period, especially for children and adolescents [39]. The link between psychological factors and cardiovascular health has been long studied. Epidemiological data show the effect of negative psychological states on the cardiovascular system, linking depression, perceived stress, anxiety, and hostility to increased cardiovascular risk [40]. Stress influences circulating inflammatory markers, and these effects may mediate the influence of psychosocial factors on cardiovascular risk [41]. In a review study, Steptoe et al. showed robust effects for increased levels of circulating of IL-6 and IL-1beta following acute stress, and marginal effects for C-reactive e protein. A number of psychobiological mechanisms may underlie responses, including stress-induced reductions in plasma volume, upregulation of synthesis, or enlargement of the cell pool contributing to synthesis [41]. Acute systemic inflammation leads to a temporary increase in large-artery stiffness in healthy populations [42]. Recent study shows that early life adversity, depression, and obesity are associated with increases in low-grade inflammation in adolescents [43]. Chronic inflammation has been shown to have a role in pathogenesis of atherosclerosis [44].

The pandemic has drastically affected the routine and habits of the general population [1, 3]. The decrease in the practice of physical activities or sedentary lifestyle, associated with harmful eating behaviours, has already been demonstrated in children and adolescents, which increased the risk of obesity [45]. In our study, weight and BMI did not differ significantly between groups. In contrast, the HC was significantly higher in the APG, without, however, any difference in the WC/HC or WHtR indices that are used as predictors of metabolic and cardiovascular alterations [46]. Similar to our study, Rúa-Alonso et al. [47] compared the body composition of two independent samples of children and adolescents obtained from a database before and during the COVID-19 pandemic. These authors observed higher values of BMI, WC, HC, and WHtR in the post-pandemic group. In a longitudinal study, Jarnig et al. evaluated 708 children aged 7 to 10 years before and during the pandemic and found increased WC/HC and WHtR [48]. BMI has also increased significantly during the pandemic and as a result, the number of overweight, obese, or extremely obese children has increased from 15.0 to 21.2%, representing a relative increase of 41.3%. In another longitudinal study, Ramos-Álvarez et al. observed an increase in BMI and percentage of fat in children aged 11 and 12 years, with no change in WC values in the post-pandemic period [49]. These studies show that there is still no consensus in the literature regarding the worsening of anthropometric values. In addition, many of these studies focused only on assessing anthropometric data and did not assess baseline physical activity level in the two periods studied. In our study, we showed a worsening in the baseline physical activity level, with an increase in the number of children considered sedentary and a decrease in the number of children considered active according to the IPAQ-C. However, despite a clear trend observed in the worsening of baseline physical activity level, we did not observe a significant difference between APG and BPG, which could be related to the reduced sample size of our study.

When comparing the QoL domains between the study groups, we observed a significant worsening in the emotional and school domains in the APG in relation to the BPG. The worsening in these domains was expected as the impact of the COVID-19 pandemic on the mental health of children and adolescents has been demonstrated in numerous studies [5, 50, 51]. During COVID-19 pandemic, most parents have found themselves forced to restructure their routines to balance working from home with parental responsibilities. Therefore, this sudden overload has subjected parents to stressful situations, potentially increasing the risk of emotional and behavioral problems in children [52]. In addition, long periods of social isolation observed in COVID-19 pandemic well known to be associated with avoidance behaviors, a deteriorating family relationship and more conflicts at home, which can drastically impact in the quality of life, mainly in emotional domains [53]. Other studies have reported depression and anxiety in children and adolescents during Covid Pandemic, which could be related to emotional domain evaluated QoL [50, 51]. This could be attributed to social isolation and loneliness experienced by this population during the critical period of the pandemic [50, 51]. In addition, the major change in study routine imposed by the pandemic has had important repercussions on school performance [5, 54]. An impairment in self-report quality of life has been shown, especially in school and psychological domains [55].

We found two studies in the literature that correlated a worsening in QoL in children and adolescents with an increase in arterial stiffness. Santos et al. found no association between QoL in healthy children and adolescents and AIx@75 [19]. In contrast, Rossi-Monteiro et al. showed an association between worsening QoL in children aged 3 to 10 years with obstructive sleep-disordered breathing and an increase in PWV [20]. For the adult population, however, it has already been established that worsening in QoL is associated with an increase in arterial stiffness rates [56–58].

The main objective of the present study was to assess whether arterial stiffness indices, PWV and AIx@75, would be altered in the study population one year after the COVID-19 pandemic. Some studies have already evaluated non-invasive arterial stiffness markers in the young population, due to the high predictive value of this variable for the development of cardiovascular diseases. Shiraishi et al. showed that central blood pressure values measured with the Mobil-O-Graph® are accurate in children and hold promise as markers of cardiovascular risk in the paediatric population [37]. Several studies show standardisation of values of the main arterial stiffness indices in children and adolescents, both by traditional methods of measuring carotid-femoral PWV and brachial artery flow-mediated vasodilation, and by indirect systems derived from algorithms calculated from brachial artery oscillometry [19, 59, 60]. Torigoe et al., amongst others, reinforced the importance of using arterial stiffness markers in children and proposed reference equations for AIx@75 values in different clinical conditions [60]. Thus, we asked whether these markers could be altered in the paediatric population, since the major change in routine imposed by the pandemic radically changed eating habits and physical activity, which are factors clearly associated with important changes in the cardiovascular system.

In the present study, we showed that the main determinant of arterial stiffness in children, AIx@75, was significantly higher in APG participants. Since our groups were matched by sex, age and socioeconomic class, our data suggest that conditions associated with routine changes resulting from the COVID-19 pandemic, such as changes in QoL due to alterations in eating habits, emotional changes, and decreased physical activity, could lead to early changes in vascular architecture and functioning. In addition to AIx@75, PWV has been shown to be a good marker of arterial stiffness. However, some authors have shown that PWV is not an adequate marker in the paediatric population, despite being important in adults [61, 62]. Some authors even describe that AIx is not linearly associated with PWV and argue that this phenomenon can be modulated by ageing, inflammation, and increased activity of the autonomic nervous system [63]. It has been reported that in the young population, AIx increases with age, whereas aortic PWV does not [61]. This is justified because the increase in AP would be due to an increase in the magnitude and not in the velocity of the reflection wave. Thus, several studies have shown changes in AIx@75 without significant changes in PWV in children, which corroborates our findings [19, 20, 24, 26, 35]. The inverse of this process occurs in older individuals, where PWV increases and AIx changes slightly, which suggests that the increase in AP would be driven by an earlier return of the reflected wave and a less compliant aorta rather than predominant changes in the magnitude of wave reflection [18, 61]. It is expected that in situations where there is an increase in AIx, the DBPc will be reduced, as was observed in our study of the APG. This happens because the early return of the reflected wave leads to a lower DBPc in the aorta [64, 65]. The decrease in DBPc is related to lower perfusion of important vascular beds such as the brain and coronary arteries, which is speculated to cause long-term dysfunctions in these tissues [64].

In the present study, an increase in HR was observed in the APG. Wilkinson et al. demonstrated that there is an inverse and linear relationship between AIx and HR resulting from changes in the time of the reflected wave caused by reductio in the duration of systole [66]. This increase in HR observed in the APG in our study may also have attenuated the increase observed in AIx@75. This change in HR could reflect changes in the physical activity routine and emotional changes experienced by the study population. We found a single study that proposed to assess resting HR in adult individuals with worsening emotional parameters or physical activity resulting from the COVID-19 pandemic before, during, and after the lockdown [67]. These authors showed an increase in resting HR during and after imposed restriction measures [67]. We believe that this can also be extrapolated to the paediatric population and would corroborate our findings.

Another factor that may have also limited the increase in AIx@75 in the APG was the reduction in TVR. Kelly et al. demonstrated that vasodilator drugs that reduce TVR are related to a decrease in AIx@75 [68]. Another parameter that deserves attention is SV, which was reduced in the APG. However, the CO was similar in both groups since the APG presented with higher HR.

The non-observance of major anthropometric changes between the two study groups does not mean that social isolation and restrictive measures imposed by the pandemic have not actually contributed to an increase in the cardiovascular risk of children and adolescents in the study. In adult patients, anthropometric data, including BMI and abdominal and hip circumferences, are related to increased arterial stiffness and mortality from cardiovascular events [46]. However, no correlation has been observed between these anthropometric data and the increase in arterial stiffness in healthy children and adolescents [19, 60]. Thus, some studies have pointed out that anthropometric measurements of body composition do not adequately reflect cardiovascular risk in the paediatric population.

### Study strengths and limitations

Our study has several strengths that should be highlighted. First, although our comparisons were made in two independent samples, children and adolescents were matched by sex and age and socioeconomic class. Second, according to the sample calculation, 60 participants would be needed in each group and our study eventually included 89 and 104 participants in the BPG and APG, respectively. One of the limitations of the study was the sample selection. The BPG was selected from nine representative regions of a large metropolitan city and the ABG was selected from only two regions. Another limitation was the use of BMI as a measure to interpret weight variation. Body composition can be highly variable and still yield the same BMI. In addition, BMI does not provide information on the regional distribution of body fat.

Although volunteers who reported being positive for COVID-19 infection were excluded from the study, some children may have been previously infected and undiagnosed, as this type of infection shows few clinical signs in the paediatric population. This may have influenced the observed results.

## Conclusion

Our results show that routine changes resulting from the COVID-19 pandemic increased cardiovascular risk in children and adolescents during the period of social isolation, as evidenced by the increase in AIx@75, considered an important marker of cardiovascular risk in the paediatric population.

**Declarations**.

## Data Availability

The datasets analysed during the current study is available from the corresponding author on reasonable request.

## Abbreviations

AIx@75: Augmentation index normalized to a heart rate of 75 beats per minute
AP: Augmentation pressure
APG: Post-Pandemic (After the Pandemic Group)
BMI: Body mass index
BPG: Pre-Pandemic (Before the Pandemic Group)
CO: Cardiac output
DBPc: Central diastolic blood pressure
DBPp: Peripheral diastolic blood pressure
HC: Hip circumference
HR: Heart rate
IPAQ-C: Children’s Physical Activity Questionnaire
ISAAC: International Study of Asthma and Allergies in Childhood
MAPp: Peripheral mean arterial blood pressure
Peds-QL 4.0: Paediatric Quality of Life Inventory version 4.0
PPc: Central pulse pressure
PPp: Peripheral pulse pressure
PWV: Pulse wave velocity
SBPc: Central systolic blood pressure
SBPp: Peripheral systolic blood pressure
SV: Stroke volume
TVR: Total vascular resistance
UNICEF: United Nations International Children’s Emergency Fund
WC: Waist circumference
WC/HC: Waist-to-hip ratio
WHO: World Health Organization
WHtR: Waist-to-height ratio
